# Electrode Elastic Modulus as the Dominant Factor in the Capping Effect in Ferroelectric Hafnium Zirconium Oxide Thin Films

**DOI:** 10.1021/acsami.4c15934

**Published:** 2024-12-03

**Authors:** Megan K. Lenox, Md Rafiqul Islam, Md Shafkat Bin Hoque, Chloe H. Skidmore, Alejandro Salanova, Shelby S. Fields, Samantha T. Jaszewski, Jon-Paul Maria, Patrick E. Hopkins, Jon F. Ihlefeld

**Affiliations:** † Department of Materials Science and Engineering, University of Virginia, Charlottesville, Virginia 22904, United States; ‡ Department of Mechanical and Aerospace Engineering, University of Virginia, Charlottesville, Virginia 22904, United States; § Department of Materials Science and Engineering, Pennsylvania State University, University Park, Pennsylvania 16802, United States; ∥ Department of Physics, University of Virginia, Charlottesville, Virginia 22904, United States; ⊥ Charles L. Brown Department of Electrical and Computer Engineering, University of Virginia, Charlottesville, Virginia 22904, United States

**Keywords:** HZO, ferroelectric, thin film, capping effect, elastic modulus

## Abstract

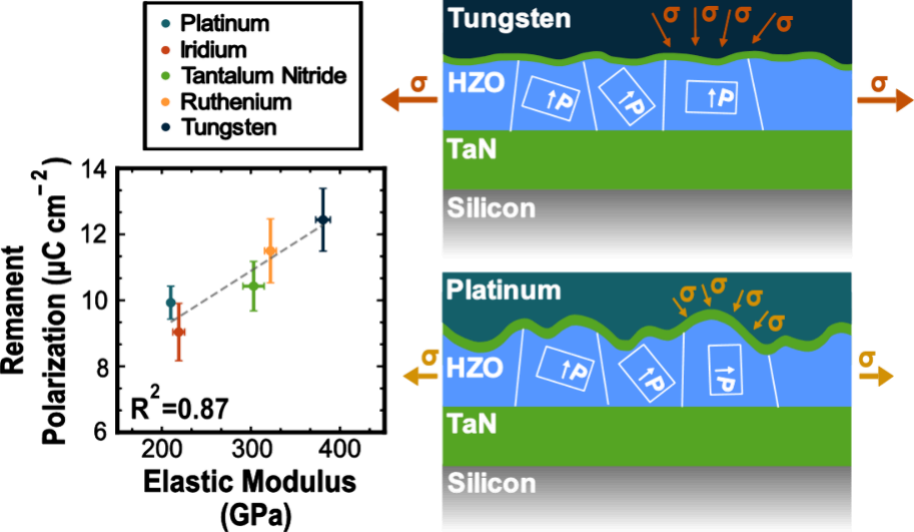

The discovery of ferroelectricity in hafnia based thin films has catalyzed significant research focused on understanding the ferroelectric property origins and means to increase stability of the ferroelectric phase. Prior studies have revealed that biaxial tensile stress via an electrode “capping effect” is a suspected ferroelectric phase stabilization mechanism. This effect is commonly reported to stem from a coefficient of thermal expansion (CTE) incongruency between the hafnia and top electrode. Despite reported correlations between ferroelectric phase fraction and electrode CTE, the thick silicon substrate dominates the mechanics and CTE-related stresses, negating any dominant contribution from an electrode CTE mismatch toward the capping effect. In this work, these discrepancies are reconciled, and the origin of these differences deriving from electrode elastic modulus, not CTE, is demonstrated. Pt/*M*/TaN/Hf_0.5_Zr_0.5_O_2_/TaN/Si devices, where *M* is platinum, TaN, iridium, tungsten, and ruthenium, were fabricated. Sin^2^(ψ)-based X-ray diffraction measurements of biaxial stress in the HZO layer reveal a strong correlation between biaxial stress, remanent polarization, and electrode elastic modulus. Conversely, a low correlation exists between the electrode CTE, HZO biaxial stress, and remanent polarization. A higher elastic modulus enhances the resistance to electrode elastic deformation, which intensifies the capping effect during crystallization, and culminates in the tandem restriction of out-of-plane hafnia volume expansion and preferential orientation of the polar *c*-axis normal to the plane. These behaviors concomitantly increase the ferroelectric phase stability and polarization magnitude. This work provides electrode material selection guidelines toward the development of high-performing ferroelectric hafnia into microelectronic devices, such as nonvolatile memories.

## 1 Introduction

Energy consumption for information technologies is growing at an exponential rate, further stressed by the emergence of machine learning and artificial intelligence.^1,2^ Flattening and reducing energy demands associated with computing requires the development of new computing and memory architectures. Toward this end, compute-in-memory and non-volatile memories hold promise, and can be realized with complementary metal oxide semiconductor (CMOS) compatible ferroelectric-based devices. While ferroelectric random access memories (FeRAM) have been commercially available for over two decades, the ferroelectrics used (lead zirconate titanate and strontium bismuth tantalate) do not scale well to nanometer thicknesses and are not thermodynamically compatible with mainstream semiconductors, such as silicon. As a result of these limitations, FeRAM has been limited in size, density, and application space.

The emergence of ferroelectricity in oxides and nitrides with fluorite and wurtzite crystal structures,^3,4^ for which the compositions and processing conditions are compatible with CMOS, has reinvigorated the pursuit of scaled ferroelectric memories.^5−7^ Recently, Ramaswamy et al.^8^ reported a 32 Gb nonvolatile dynamic random access memory (NVDRAM) utilizing fluorite-structured ferroelectric hafnium zirconium oxide (HZO) with a 10 year data retention from −40 to 95 °C and 10^12^ cycling endurance, exemplifying the promise of HZO in next generation scaled memories.^9^ This success provides evidence that ferroelectrics based upon HZO and other doped hafnia compositions have undergone significant development in both device applications and scientific understanding of the origin of the ferroelectric response. For example, the ferroelectric response is now well established in non-epitaxial films to originate from a metastable orthorhombic phase with a *Pca*2_1_ space group, denoted as the o-III phase.^10^ While it does not appear on any equilibrium phase diagram, its stability has been attributed to many factors, including local differences due to dopant ionic radii,^11,12^ tensile stresses,^13−15^ reduced surface/interfacial energy compared to equilibrium phases,^16,17^ and oxygen vacancies.^18−21^ Tailoring compositions, film thickness, and oxygen content are now recognized methods with which to control phase content.

Processing conditions and adjacent electrodes are also known to affect phase formation and stability. In the first report of ferroelectricity in hafnia,^3^ and the majority of subsequent studies, greater volume fractions of the o-III phase were acquired when post-metallization annealing (PMA) was used to crystallize the hafnia layer with a top electrode in place. Multiple mechanisms have been invoked to explain this result. First, commonly used metal nitride electrodes, such as TiN and TaN, can preferentially scavenge oxygen from hafnia, generating interfacial layers, and subsequently increase oxygen vacancy concentrations in the hafnia required for o-III phase stabilization.^18,22,23^ Second, electrodes have also been correlated with inducing a tensile biaxial stress upon cooling when annealed in a PMA procedure.^24^ This tensile stress has two benefits toward a greater polarization response: tensile stresses have been computationally predicted to drive the formation of the o-III phase from the parent tetragonal phase^15^ and tensile stress will favor the orientation of the intermediate length polar *c*-axis normal to the film surface.^12^

The origin of the large residual stress in ferroelectric hafnia films has previously been postulated to result from a coalescence of islands during growth,^24^ densification during the crystallization anneal,^25^ lattice strain from substrate lattice mismatch during epitaxial growth,^26^ thermal expansion mismatch with the substrate,^13^ and thermal expansion mismatch stress induced by low coefficient of thermal expansion (CTE) electrodes during the PMA process.^14^ With respect to electrodes, Mueller et al. noted that PMA processed films possessed lower roughness values than their non-electroded counterparts and suggested that out-of-plane displacement may be affected by the electrode.^27^ Expounding upon this, Fields et al. established that the capping effect is due to the electrode membrane force, which prevents an out-of-plane displacement and formation of the monoclinic phase. In hafnia and HZO, the monoclinic phase possesses a larger molar volume than the metastable tetragonal, o-III, and antipolar orthorhombic (*Pbca*, o-I) phases.^17^ Monoclinic phase nucleation from any of these metastable phases requires a 3-dimensional expansion, which is inhibited by the membrane force imposed by the top electrode. Thus, the hafnia layers retain the large stress resulting from the other mechanisms (densification, island coalescence, and thermal expansion mismatch with the substrate) because the electrode is in place. In fact, the top electrode cannot impart significant stress in an underlying film as noted by Chernikova et al., who estimated an electrode-induced tensile stress of only 155 MPa,^28^ and Ihlefeld et al., who estimated an electrode-induced compressive stress of 2 MPa.^29^ This notwithstanding, there are many observations of differences in ferroelectric phase content and measured polarization magnitude for hafnia-based films prepared with electrodes of differing CTEs.^30−32^ Therefore, there exists a knowledge gap explaining why different electrodes result in differing o-III phase fractions and polarization magnitudes in hafnia-based films.

In this work, the origin of the capping effect with respect to top electrode elastic modulus (*E*) is investigated in HZO thin films and capacitors. The interatomic potential that dictates a material’s CTE also drives its elastic modulus, leading to a general relationship between 1/CTE and *E*. A material with a higher bond strength typically has a diminished response to external stimuli such as temperature and stress resulting in a low CTE and high *E*. The converse is typically true of low bond strength materials, with a low *E* and high CTE. To study the electrode modulus effect, five electrode materials with varying CTE and elastic moduli values were selected: tungsten, TaN, platinum, iridium, and ruthenium. The results will show that electrode CTE does not correlate well with ferroelectric performance. However, there is a direct correlation of electrode elastic modulus with the stress within the HZO layer and its remanent polarization, which is due to an increased impediment to out-of-plane displacement of the HZO layer with increased electrode modulus.

## 2 Results and Discussion

**Figure 1:**
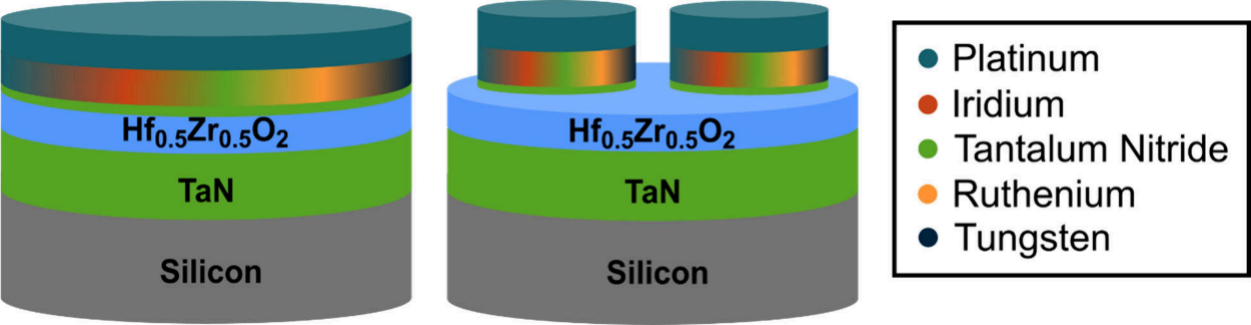
Schematic of the metal-ferroelectric-metal geometries of the devices fabricated with the investigated electrode materials. The multicolored layer represents the five different electrode materials investigated.

Film structures were prepared to minimize differences in electrode chemical interaction in contact with hafnia and stresses associated with the as-deposited condition of the electrode. To accomplish this, each sample was fabricated with a 100 nm thick sputtered TaN bottom electrode, 18.9 nm of Hf_0.5_Zr_0.5_O_2_ prepared via plasma-enhanced atomic layer deposition, 2 nm of sputtered TaN, 20 nm of the investigated electrode material deposited via sputtering, and 20 nm of sputtered platinum. All components of the top electrode stack (TaN, different electrode materials, and platinum) were deposited sequentially without breaking vacuum. The 2 nm thick TaN layer placed between the varied electrodes and HZO was used to create a chemically symmetric electrode interfacial layer to minimize possible differences in oxygen scavenging by electrode materials of differing elastic modulus. Electrode sputter conditions were determined to place each as-sputtered electrode in a compressive stress state to further minimize differences between electrodes, as shown by wafer flexure measurements in Supporting Information Figure S1. The 18.9 nm HZO layer thickness was selected to provide a large signal-to-noise ratio for X-ray diffraction sin^2^(ψ) measurements. The sputter deposited thin film electrode elastic moduli were experimentally derived from picosecond acoustics measurements of sound velocity to account for microstructural and density differences between thin film and bulk materials.^33−35^ The indexed time delay signatures for each electrode material, along with an aluminum transducer layer, are shown in Supporting Information Figure S2. Two samples of each set were prepared, with schematic depictions of the structures shown in Figure 1. One sample consisted of a blanket coverage of all layers for phase and stress analysis via X-ray diffraction and wafer-flexure based stress measurements of the entire film stack. The second sample was prepared with defined electrodes deposited via a shadow mask for electrical property measurements. The circular electrode diameters ranged from 30 to 100 μm. After top electrode deposition, samples underwent rapid thermal annealing at 600 °C in an N_2_ atmosphere for 30 s to crystallize the HZO.

The elastic moduli, literature values for CTEs, HZO biaxial stresses calculated via the sin^2^(ψ) method, as well as the pristine and awoken remanent polarization (*P*_r_) values derived from positive-up-negative-down measurements (PUND) are presented in Table 1. From the picosecond acoustic measurements, platinum and tungsten have the lowest and highest elastic moduli at approximately 210 and 380 GPa, respectively. The reported linear coefficient of thermal expansion of each electrode material, besides TaN, are tabulated, where the CTE reported is the average of the room temperature value and that at 600 °C, which is the annealing temperature used in this study.^36−39^ The linear CTE for TaN was determined from TaN powder using X-ray diffraction in a Bragg–Brentano geometry over a range of temperatures from 25 to 650 °C. This measurement was conducted owing to a lack of literature sources on this material. The TaN *d*-spacings were calculated from Rietveld refinement, which were then utilized to derive the linear CTE. The temperature dependent *d*-spacings and CTE data are provided in the Supporting Information and in the University of Virginia Dataverse.^40^ Platinum and tungsten have the largest and smallest reported CTE values of electrode materials investigated in this study, at 9.64 and 4.45 × 10^–6^ K^–1^, respectively. Iridium and ruthenium have similar respective CTE values of 7.45 and 7.95 × 10^–6^ K^–1^, but differ in their elastic moduli, at approximately 219 and 322 GPa. This similarity in CTE, but difference in elastic modulus will aid in decoupling CTE and elastic modulus effects on the capping effect.

**Table 1:** Measured Elastic Modulus (*E*), Linear Coefficient of Thermal Expansion (CTE), Biaxial Stress in the HZO Layer (σ), and Pristine and Awoken Remanent Polarization Derived from PUND (*P*_r_)

Electrode Material	*E* (GPa)	Linear CTE (×10^–6^ K^–1^)	sin^2^(ψ) HZO σ (GPa)	Pristine *P*_r_ (μC cm^–2^)	Awoken *P*_r_ (μC cm^–2^)
Platinum	210.4 ± 1.91	9.64^36^	3.56 ± 0.1	6.43 ± 0.33	9.93 ± 0.50
Iridium	219.4 ± 6.57	7.45^37^	3.80 ± 0.3	6.33 ± 0.89	9.04 ± 0.87
Tantalum Nitride	303.4 ± 11.9	5.50	3.85 ± 0.2	6.93 ± 0.15	10.43 ± 0.75
Ruthenium	322.3 ± 6.57	7.95^38^	4.00 ± 0.3	7.73 ± 0.24	11.50 ± 0.97
Tungsten	381.8 ± 8.17	4.45^39^	4.21 ± 0.3	9.55 ± 1.70	12.44 ± 0.95

To quantify the HZO layer biaxial stress for each sample, the X-ray diffraction sin^2^(ψ) method was utilized with a previously determined elastic modulus for HZO.^41^ Differences in elastic modulus with phase were shown in the prior work to be less than 5% and would lead to small errors in reported stress values. Stylus profilometry was used to monitor the wafer flexure stress of the film stacks at each processing step, beginning with bare silicon wafers. Profilometry data was then fit using a polynomial best fit equation to arrive at the radius of curvature of each layer. The stress thickness product of each layer and of the composite with respect to each processing step was then calculated and is plotted in Supporting Information Figure S1, where it is confirmed the deposition of the top electrode induced a comparable compressive stress to each sample. Remanent polarization was measured using a PUND technique employing a 2.5 MV cm^–1^ field 1 kHz pulse wave with a 100 ms delay and 1 ms pulse duration.

**Figure 2:**
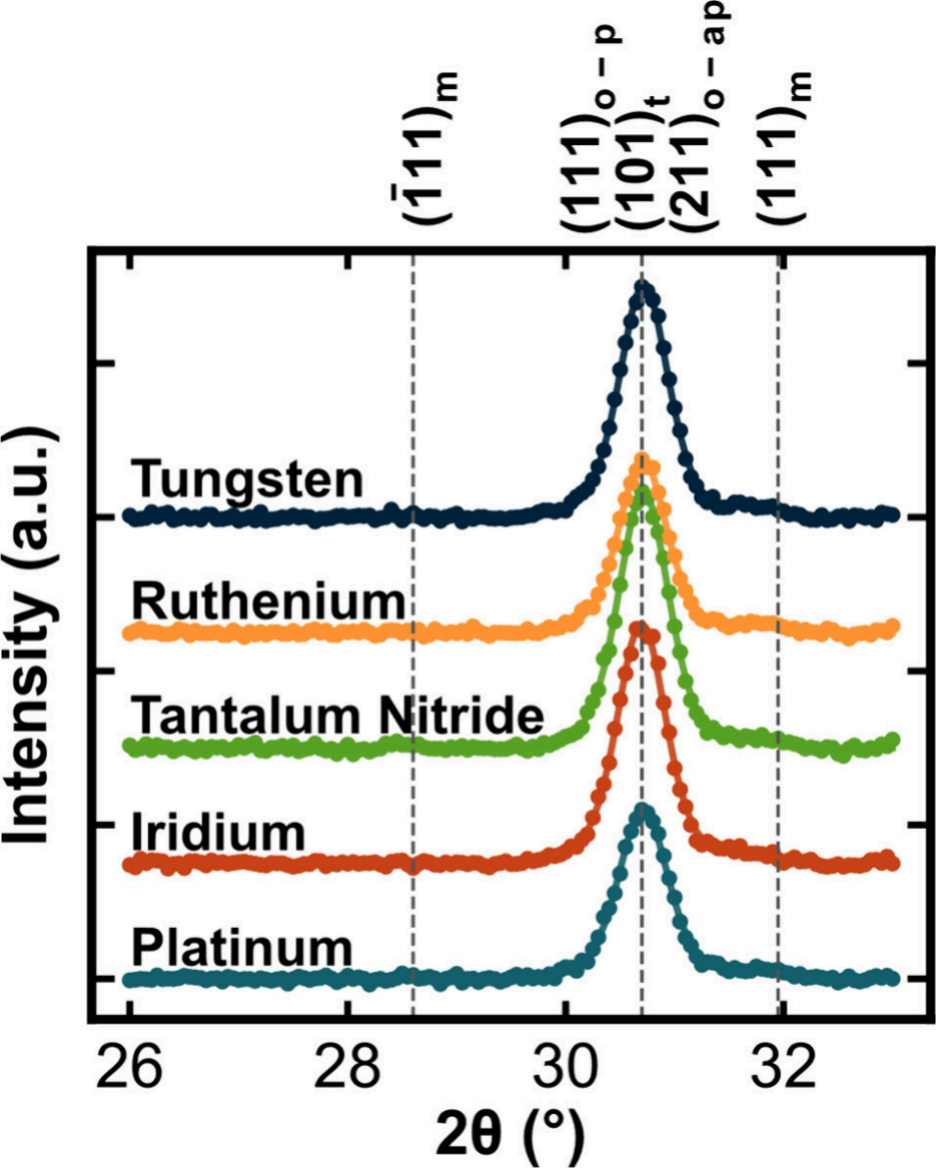
Grazing incidence X-ray diffraction patterns taken from 26 to 33° 2θ angle, with indexing of reflections characteristic of the equilibrium monoclinic phase at 28.5 and 31.8°, and nonequilibrium o-III, o-I, and tetragonal phases at nominally 30.4° for samples prepared with each of the investigated top electrode materials.

Grazing-incidence X-ray diffraction (GI-XRD) patterns focusing on the most intense HZO reflection range are shown in Figure 2 for each film. Note that the colors used for each different electrode are consistent throughout all figures in this manuscript. The positions of the HZO monoclinic (111) and (111) peaks at 28.5 and 31.8° and the nonequilibrium phase peak at ∼30.4° are denoted with vertical dashed lines. It is not possible to differentiate the nonequilibrium o-III (*Pca*2_1_), o-I (*Pbca*), and tetragonal phases via GI-XRD owing to the similarity of their *d*-spacings, the low intensities of otherwise distinguishing reflections, and the broad peak widths owing to small film thickness and grain sizes. Therefore, this reflection at ∼30.4° will be denoted as the metastable phase. The GI-XRD patterns were fit using LIPRAS^42^ and reveal no measurable monoclinic phase present or peak shift of the metastable phase reflection within detection limits for these films. This indicates that a comparable fraction of nonequilibrium phases is present and, within the limitations of lab-scale GI-XRD, the differing top electrode moduli and CTE did not impart significant differences in the phase content. The absence of the monoclinic phase is also indicative of the capping effect of the top electrode constraining the out-of-plane displacement of the films, thus impinging the volume expansion required to form the monoclinic phase.

**Figure 3:**
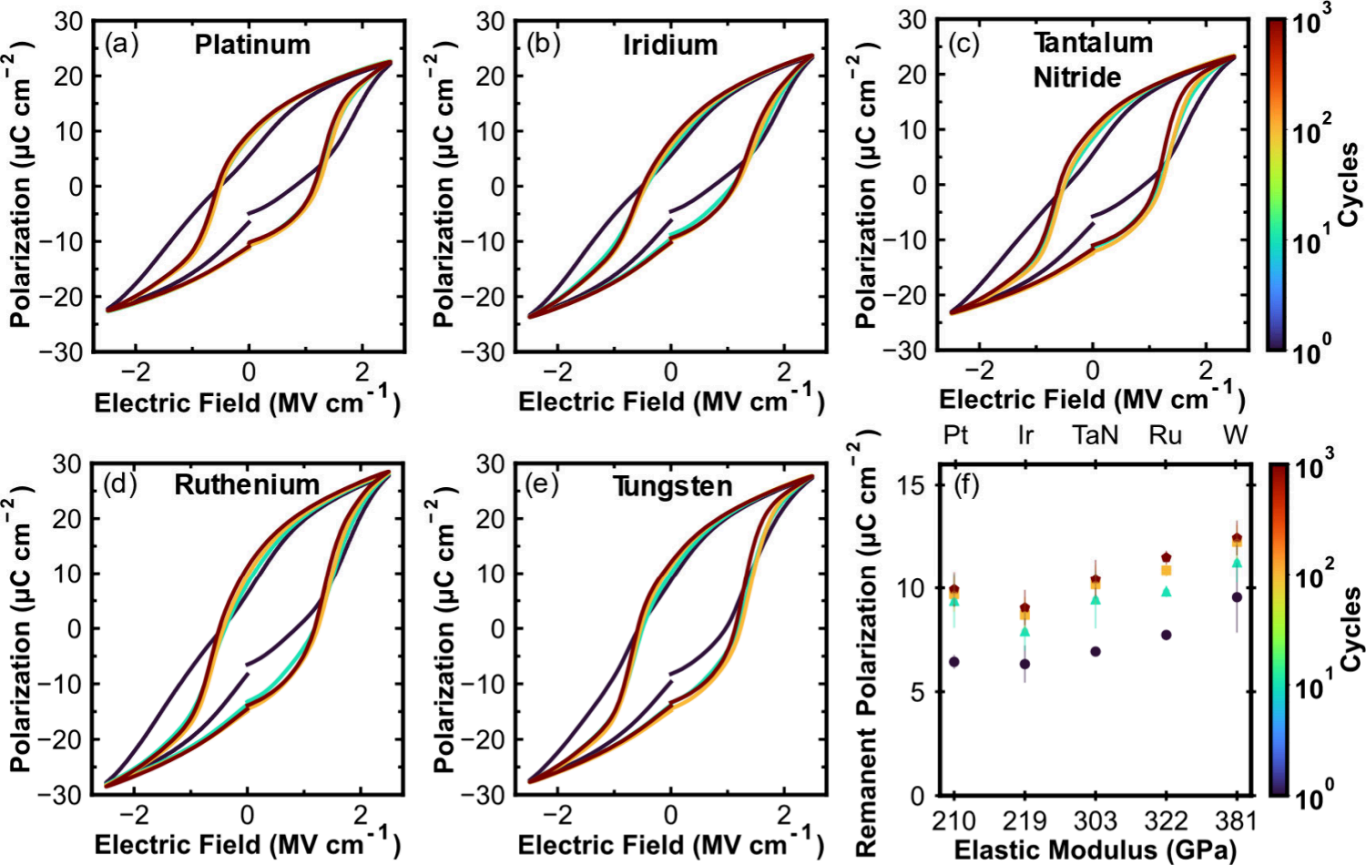
Measured *P*(*E*) loops taken at 2.5 MV cm^–1^ field at decade intervals from the pristine state to 10^3^ cycles using a 2.0 MV cm^–1^ field 1 kHz square cycling wave for (a) platinum, (b) iridium, (c) TaN, (d) ruthenium, and (e) tungsten, respectively. (f) Remanent polarization measured from PUND measurements taken at each cycling interval and plotted against the measured elastic modulus of each material. The color bar on the right indicates the number of field cycles.

In contrast to the GI-XRD results, the *P*(*E*) responses shown in Figure 3a–e reveal clear effects of the top electrode on the HZO polarization behavior. *P*(*E*) behaviors were measured with a 2.5 MV cm^–1^ maximum electric field from the pristine state. Devices were then field cycled at a 2.0 MV cm^–1^ field using a 1 kHz, 50% duty cycle square wave applied in decade intervals (e.g., 10^1^, 10^2^, and 10^3^) until 10^3^ cycles, with *P*(*E*) measurements taken at each interval. With field cycling, there is an accompanying depinching of the hysteresis response and an increase in the remanent polarization for all devices. These phenomena are characteristic of wake-up and indicate either a transformation from the antipolar o-I phase to the ferroelectric o-III phase,^43−45^ a transformation from the tetragonal phase to the o-III phase,^46,47^ ferroelastic switching resulting in the short polar *c*-axis transitioning from an in-plane to out-of-plane orientation,^48,49^ or a breakdown in a non-ferroelectric interfacial layer.^50,51^ It is noted that devices show minimal leakage current at the saturation polarization (*P*_s_) for all conditions. Despite the multiple possible wake-up mechanisms, each sample shows a consistent degree of wake-up with field cycling, with a roughly 45% increase in *P*_r_ for each sample. This suggests that the responsible mechanisms for wake-up occur in all samples to a similar degree. Figure 3f shows PUND measurements taken following *P*(*E*) loop measurements at each field cycling interval and plotted with respect to electrode material and measured elastic modulus. A clear trend between measured *P*_r_ and elastic modulus is observed. The lowest modulus electrode, platinum, results in the lowest measured *P*_r_. The highest modulus electrode, tungsten, results in the highest measured *P*_r_. It is observed that the dependence of *P*_r_ on electrode modulus is maintained from the pristine state through the woken-up state.

**Figure 4:**
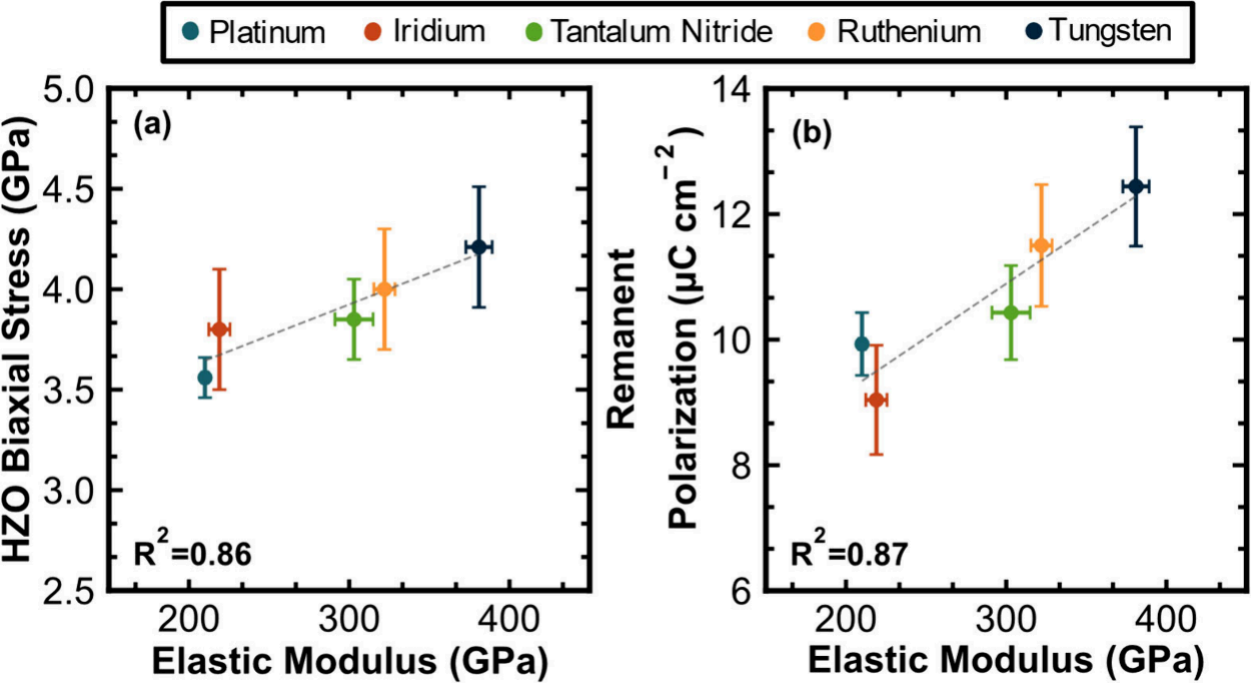
(a) Sin^2^(ψ)-derived HZO biaxial stress plotted versus the measured top electrode elastic modulus for each electrode material. (b) Awoken *P*_r_ extracted from PUND versus the top electrode elastic modulus.

To expand upon the correlative effects of the electrode elastic modulus and how it affects the HZO layer, the electrode elastic modulus is plotted against the HZO biaxial stress in Figure 4a. The sin^2^(ψ) plots used to derive the HZO biaxial stress are in Supporting Information Figure S3. A monotonic trend exists between capping electrode elastic modulus and the degree of biaxial stress induced in the HZO film. A linear regression was performed on the dependence and resulted in a 0.86 coefficient of determination (*R*^2^) value. Figure 4b compares the electrode elastic modulus to the HZO remanent polarization. As with the biaxial stress, there is a clear linear dependence of electrode elastic modulus and the remanent polarization. An *R*^2^ value of 0.87 is obtained from a linear regression.

When annealing without a top electrode present, a volume expansion is free to occur along all directions, fostering the transition from lower molar volume metastable phases to the higher molar volume equilibrium monoclinic phase. As previously stated, during PMA, the top electrode creates a membrane force at the HZO interface, impeding volume expansion in the direction normal to the surface.^25^ Consider platinum and tungsten, which, respectively, have the lowest (210 GPa) and highest (382 GPa) elastic moduli among the electrodes used in this study. Tungsten exhibits greater resistance to elastic deformation, preventing the 3-dimensional expansion. In turn, this resistance to expansion drives the persistence of tensile stresses that develop in the HZO layer due to densification and the thermal expansion mismatch with the thick silicon substrate. The capping effect, controlled by the top electrode elastic modulus, is analogously measured through the biaxial stress in the film. Platinum is less resistant to elastic deformation and results in HZO with a biaxial tensile stress of 3.56 GPa. A tungsten electrode, in contrast, provides the greatest resistance to elastic deformation of the electrodes investigated in this study, and results in the HZO layer having a biaxial tensile stress of 4.21 GPa. Further, the greater tensile stress induced by the capping effect may promote a preferred ferroelastic texture where the shorter polar *c*-axis, as opposed to the long non-polar *a*-axis, is normal to the plane, resulting in a greater portion of domains in the polycrystalline film that can contribute to the polarization response. Orienting the long *a*-axis out of plane would require a local roughening at the top surface of the HZO layer, which would require a local deformation of the top electrode. A high modulus electrode would resist this deformation more than a low modulus electrode. Similarly, orienting along the *b*-axis, with a similar but smaller lattice spacing to the *c*-axis could also be preferred. However, this ferroelastic texturing would reduce the overall portion of domains that can contribute to the polarization response, which is not observed.

**Figure 5:**
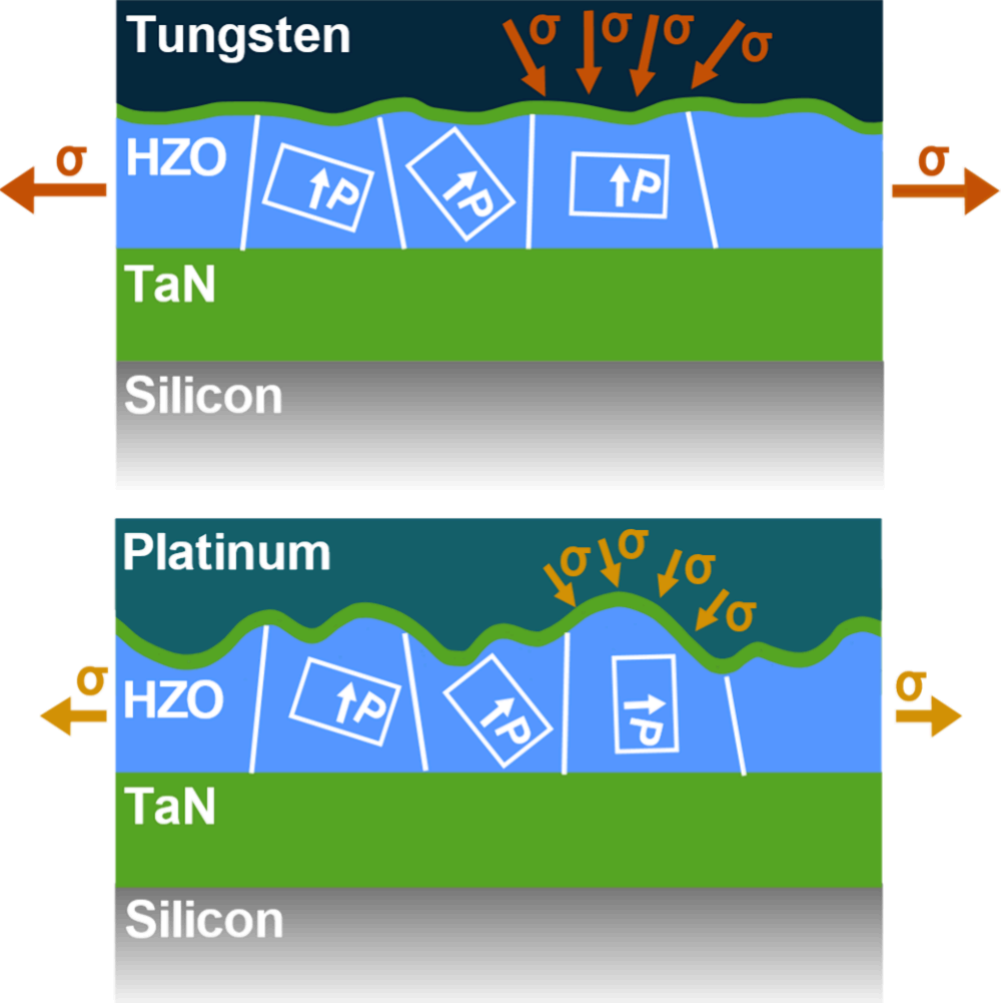
Schematic depiction of devices fabricated with a tungsten (larger *E*) top electrode exhibiting a greater capping effect, resulting in more significant biaxial stress within the HZO layer and concomitant preferential *c*-axis out-of-plane o-III phase ferroelastic texture, and a platinum top electrode (lesser *E*) resulting in an opposing trend. The length of the arrows depicts the magnitude of stresses.

Figure 5 illustrates the ferroelastic texturing model. The model is supported by considering that devices fabricated with a large elastic modulus tungsten electrode have an awoken remanent polarization of ∼12.44 μC cm^–2^, while a small elastic modulus platinum electrode resulted in a *P*_r_ of ∼10 μC cm^–2^ while no observable difference in the phase makeup of these films could be discerned. The concomitant increase in both HZO stress and remanent polarization highlights the impact of electrode elastic modulus on the capping effect imparted to the HZO layer and the generation of robust ferroelectric HZO based devices.

**Figure 6:**
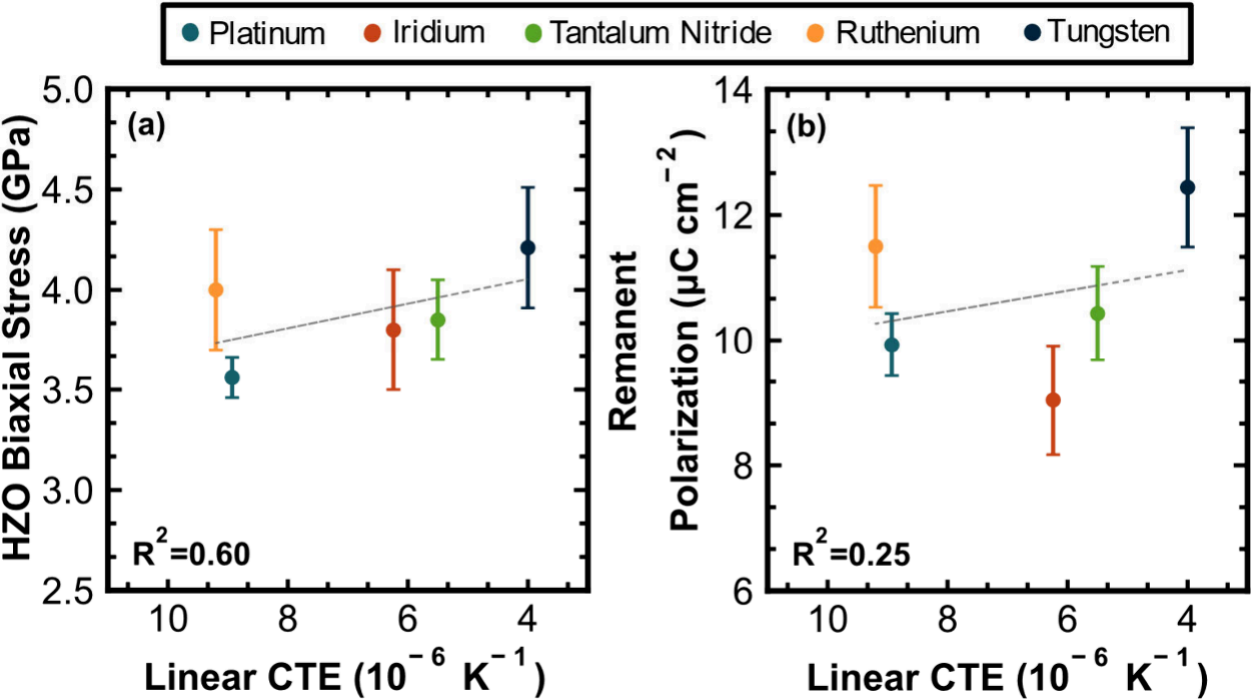
(a) Sin^2^(ψ) HZO biaxial stress plotted versus reported^36−39^ and measured linear CTE values for each electrode material. (b) Awoken *P*_r_ versus top electrode linear CTE.

In contrast, the same comparisons can be made between electrode linear CTE, HZO biaxial stress, and awoken *P*_r_. These results are shown in Figure 6a and b, respectively. The HZO biaxial stress reveals a weak statistical correlation with electrode linear CTE, as demonstrated by a linear regression *R*^2^ value of 0.60. Similarly, the HZO *P*_r_, shown in Figure 6b, also exhibits a weak correlation with CTE, as characterized by a linear regression *R*^2^ value of 0.25. These results support the prior calculations from Chernikova et al.^28^ and Ihlefeld et al.^29^ who both calculated that top electrode CTE provides a negligible contribution to the capping effect. To demonstrate this, iridium and ruthenium contacts can be used to compare elastic modulus and CTE effects as they have similar respective CTE values of 7.45 × 10^–6^ and 7.95 × 10^–6^ K^–1^, but vastly different respective elastic moduli of 219 and 322 GPa. If electrode CTE were the dominant factor of the capping effect, it would be expected that the stress within the HZO layer and the *P*_r_ should be similar for films and devices prepared with these two electrodes. It is evident in comparing the respective data for samples prepared with these two electrodes in Figure 4 that both the stress within the HZO layer and *P*_r_ differ greatly. The ruthenium electrode devices have higher HZO biaxial stress and remanent polarizations than the iridium electrode devices. Another comparison to illustrate the impact of elastic modulus compared to CTE on resulting HZO stress and polarization is with platinum and iridium. For these two electrodes, similar elastic moduli have been measured, at 210 and 219, respectively, but the CTEs are 9.64 × 10^–6^ and 7.45 × 10^–6^ K^–1^, respectively. Both the HZO stress and remanent polarizations are similar for devices prepared with these two metal electrodes. This cannot be reconciled by the model of electrode CTE driving the capping effect, as it would be expected that the lower CTE of iridium would lead to larger HZO stress and higher remanent polarization.

**Figure 7:**
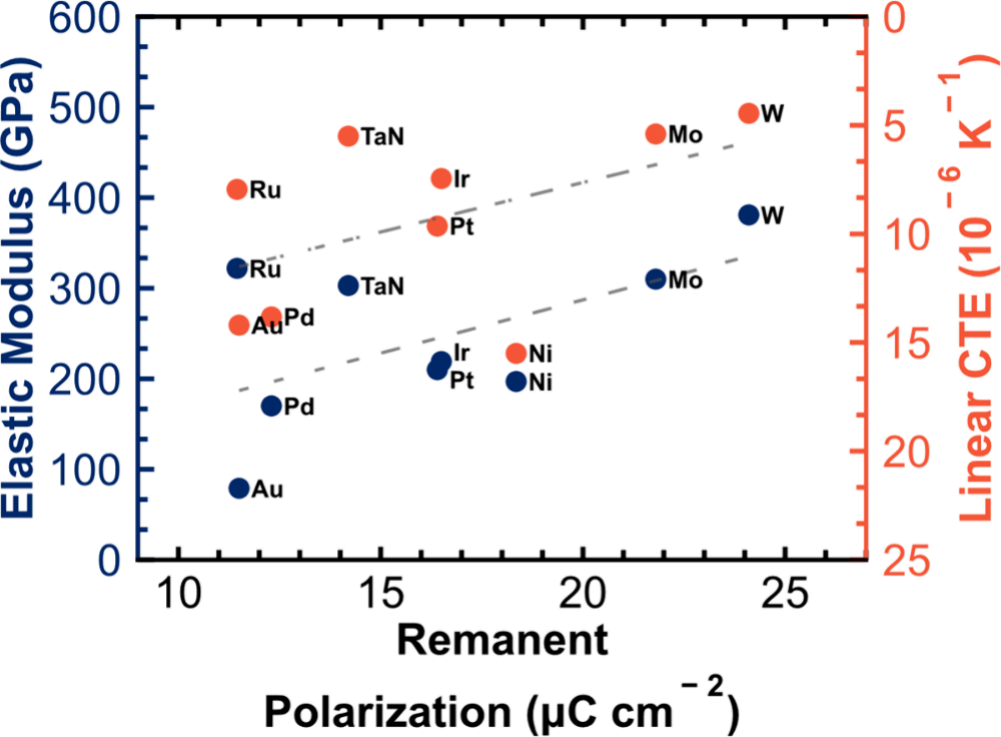
Compilation of reported remanent polarization values of devices fabricated with differing top electrode materials, plotted with respect to the electrode elastic moduli and linear CTEs.^30,31,36−39,52−62^

This work presents a lack of correlation between top electrode CTE and HZO *P*_r_ that is contradictory to results frequently reported in literature; researchers have attributed increased volume fractions of the ferroelectric phase and *P*_r_ values to an electrode CTE-induced stress within the HZO layer. Figure 7 compiles remanent polarization values for HZO samples prepared with different top electrodes as reported in literature from multiple research groups.^30,31,52−54^ The data is plotted with respect to the literature elastic modulus and CTE values of the top electrode materials.^36−39,55−62^ Both the elastic modulus and CTE have similar apparent correlations with reported *P*_r_. While it is has been shown that CTE mismatch cannot contribute meaningfully to the capping effect, these results do not discredit an effect of the electrode material and the efficacy of the capping effect contributing toward an increase in *P*_r_. The gap in understanding of why this trend exists is filled by the consideration of the top electrode elastic modulus generating a rigid membrane force necessary to impinge out-of-plane volume expansion and promote both stabilization of small molar volume phases, such as the o-III ferroelectric phase and a preferred *c*-axis out-of-plane ferroelastic texture, as depicted in Figure 5. That there is a weak correlation with CTE, as observed from the literature reports in Figure 7, may be expected. As mentioned in the introduction, in very general terms, large CTE values are often present in low bond strength materials. Concomitantly, low elastic moduli are observed when the bond strength is low. Therefore, it is reasonable to expect that a high CTE metal will lead to a poor capping effect because it will likely have a low elastic modulus. The results of this study can also reconcile the observation of Kim et al.^14^ of an increased polarization in the HZO film when the TiN capping electrode thickness was increased. A larger thickness electrode will provide a thicker membrane that is more resistant to deformation for a given force than a thin electrode and can serve as a more robust capping layer.

## 3 Conclusions

Pt/*M*/TaN/H_0.5_Zr_0.5_O_2_/TaN/Si devices, where *M* is iridium, platinum, tantalum nitride, ruthenium, or tungsten, were fabricated to investigate the effect of varying electrode elastic modulus and coefficient of thermal expansion on the HZO phase and ferroelectric performance. Elastic modulus was measured using picosecond acoustics for electrode films with the same deposition parameters used in device fabrication. Grazing incidence X-ray diffraction showed a comparable phase constitution within the HZO layer regardless of the electrode material, with all samples comprising metastable phases. The biaxial stress in the HZO layer increased with higher electrode elastic modulus. Further, a monotonic increase in the remanent polarization with electrode elastic modulus was observed. This trend persisted in both the pristine and awoken *P*_r_ values measured in devices. A low correlation was observed between the top electrode linear coefficient of thermal expansion and the HZO biaxial stress and *P*_r_. These results indicate that the electrode-dependent capping effect originates from the elastic modulus of the top electrode. Furthermore, these results promote the use of high elastic modulus metals, such as tungsten, as promising candidates for electrode materials in the development of ferroelectric hafnia-based devices.

## 4 Experimental Methods

### Electrode Deposition Conditions

Deposition processes were developed for each electrode to achieve a similar as-deposited stress state. To accomplish this, a Bruker Dektak-XT stylus profilometer was used to measure the curvature of individual 50.8 mm diameter, (111)-oriented, 280 μm thick silicon wafers. Electrode materials were then DC magnetron sputtered onto these silicon wafers using either a magnetically balanced Meivac MAK 50.4 mm diameter sputter gun (TaN, Pt, W, and Ru) or an AJA International ST10 25.4 mm diameter sputter gun (Ir) within a custom sputter deposition system using various argon gas pressure and target power conditions. Film thicknesses were measured using X-ray reflectivity with a PanAlytical Empyrean X-ray diffractometer with a Cu Kα radiation source. Wafer flexure measurements were repeated after electrode material layer deposition to calculate the radius of curvature. The stress in the electrode layer was then determined using the Stoney equation. Processing parameters were varied until all deposited electrode layers were in a compressive state as-deposited. The following processing conditions were used: tungsten (3.3 W cm^–2^ and 5 mTorr argon), ruthenium (3.3 W cm^–2^ and 10 mTorr argon), iridium (6.1 W cm^–2^ and 40 mTorr argon), TaN (3.3 W cm^–2^ and 7.5 mTorr argon), and platinum (3.3 W cm^–2^ and 5 mTorr argon).

### Sample Fabrication

Pt/*M*/TaN/Hf_0.5_Zr_0.5_O_2_/TaN/Si devices, where *M* = platinum, iridium, ruthenium, tantalum nitride, and platinum electrode materials, were fabricated. 100 nm thick TaN was sputtered from a sintered TaN target onto (001)-oriented, 500 μm thick silicon and (111)-oriented, 25.4 mm diameter, 280 μm thick silicon wafer substrates. The sputtering conditions described above were used. Plasma-enhanced atomic layer deposition (PE-ALD) was used to deposit nominally 18.94 nm of hafnium zirconium oxide using an Oxford FlexAL II instrument with a substrate table temperature of 260 °C. Tetrakis(ethylmethylamido)-hafnium (TEMA-Hf) and tetrakis(ethylmethylamido)-zirconium (TEMA-Zr) were used as Hf and Zr precursors, respectively, and were deposited in a 3:2 cycling ratio with 32 supercycles to achieve an Hf_0.5_Zr_0.5_O_2_ nominal composition and the desired thickness. 250 and 300 W respective oxygen plasma powers were used as the oxygen source for HfO_2_ and ZrO_2_ layers. The top electrodes were sputter deposited via DC magnetron sputtering of 2 nm of TaN followed by 20 nm of each “*M*” top electrode and 20 nm of platinum all without breaking vacuum. The sputter conditions for each metal are described above. Samples receiving a blanket electrode layer across entire surface were placed atop a mask platen, while samples for electrical measurements were secured under a shadow mask within the mask platen to prepare contacts of known diameter between 30 and 100 μm. This shadow masking approach was required as chemical etch procedures were not available for all electrode metals. An Allwin21 AccuThermo AW 610 rapid thermal processor was used to crystallize the HZO films at 600 °C using a 50 °C s^–1^ ramp rate in 1 atm of dynamic N_2_ flow (10 slm) for 30 s.

### Structural Characterization

Stylus profilometry using a Bruker Dektak-XT was performed on 25.4 mm diameter (111)-oriented 280 μm thick silicon wafers to monitor overall stress development following each processing step. Profilometry data was then fit using a polynomial best fit equation to arrive at the radius of curvature of each layer. The radius of curvature was input into the Stoney equation, eq 1, to calculate the stress in the film layer with respect to both the previous layer and the cumulative film at each processing step, where *E*_s_ is the elastic modulus of the substrate, *h*_s_ is the substrate thickness, ν_s_ is substrate Poisson’s ratio, *R*_F_ and *R*_0_ are the radius of curvature of the calculated and previous layers, respectively, and *h*_f_ is the thickness of the considered layer.^63^
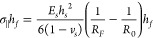
1

The HZO film phases were characterized with grazing-incidence X-ray diffraction over the 2θ range of 26–33° with a Rigaku SmartLab diffractometer with Cu Kα radiation and a fixed 1° omega angle. The LIPRAS software^42^ package was then used to fit the patterns using Pearson VII peak shapes. HZO thickness was measured using X-ray reflectivity (XRR) using a Rigaku SmartLab diffractometer with Cu Kα radiation from 0 to 6° in 2θ range. The resulting pattern was fit using GSAS-II software.^64^ Microfocus X-ray diffraction was utilized to measure biaxial stress of the HZO with a Bruker D8 Venture diffractometer equipped with an Incoatec IμS 3.0 Cu Kα source and Photon III 2-dimensional detector. Samples were prepared by adhering a thin layer of MgO powder to the surface of a blanket top electrode sample. MgO powder serves dual purposes: 1) to function as a standard for height alignment and 2) for sample displacement correction during pattern unwarping. The omega angle was set at 18° and the Photon III detector centered at 40° in 2θ. Ten 10 min scans were compiled to increase the signal-to-noise ratio. Using PyFAI software,^65^ subsequent scans were unwarped to generate an integrated 1D scan. Averaged and unwarped area detector patterns are plotted in Supporting Information Figure S4. LIPRAS software was used to fit Pearson VII shapes to patterns at varying ψ angles. The sin^2^(ψ) method was employed to arrive at the biaxial stress in the HZO resulting from strain using the change in *d*-spacing of the nonequilibrium phase peak with respect to varying ψ angles and eqs 2 and 3
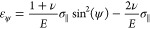
2
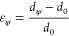
3where *d*_*ψ*_ is the *d*-spacing at each ψ angle, *v* is Poisson’s ratio (0.29 for HZO),^66,67^*E* is the HZO elastic modulus,^41^ and σ_∥_ is the biaxial stress.^25,68^

### Electrical Characterization

To measure the polarization response, the drive and return connections of a Radiant Technologies Precision LC II ferroelectric tester were connected to pristine 100 μm-diameter capacitors and the bottom electrode, respectively. Polarization hysteresis (*P*(*E*)) measurements were conducted at 2.5 MV cm^–1^ field with a 1 ms period. Pulsed positive-up-negative-down (PUND) measurements were performed at 2.5 MV cm^–1^ electric field, 1 kHz frequency, 1 ms pulse time, and 100 ms pulse delay on pristine devices. Capacitors were then field cycled using a 2 MV cm^–1^ 50% duty cycle square wave with 1 kHz frequency with *P*(*E*) and PUND measurements taken at 10, 100, and 1000 cycles to wake up devices.

### Electrode Modulus and CTE Characterization

The picosecond acoustics technique^33,34^ was performed using a time domain thermoreflectance (TDTR) setup. TDTR is a pump–probe system that uses an ultrafast (subpicosecond) pump pulse to deliver energy to the surface of a sample, and a time delayed probe pulse to monitor the change in the sample surface’s reflectivity due to the pump excitation. Upon absorption of the pump probe, the change in energy density in the absorbed volume leads to local thermal expansion and the launching of a strain wave that propagates at the speed of sound longitudinally through the thickness of the sample. This strain wave is partially reflected and partially transmitted at material interfaces. In the experiments conducted in this work, the round-trip time of the reflected strain wave launched from the metal surface and reflected off the metal/HZO interface was monitored via signatures in the probe beam’s thermoreflectivity. The longitudinal sound speed, calculated using *v*_L_ = 2*d*/τ, where *d* is the film thickness and τ is the time delay (i.e., the measured round trip time), was used in eq 4 to calculate the elastic modulus of the electrode material deposited using the same parameters employed during device fabrication, where υ is Poisson’s ratio, ρ is the film density, and *E* is the elastic modulus.
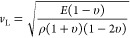
4

Data showing indexed time delay signatures for each electrode material, along with aluminum, used as a reflective top layer, is shown in Supporting Information Figure S2.

TaN coefficient of thermal expansion was measured using a Malvern Panalytical Empyrean II X-ray diffractometer with Cu Kα source and X’Celerator detector in a Bragg–Brentano geometry over a range of temperatures from 25 to 650 °C. An Anton-Paar HTK 1200N high temperature stage in an N_2_ atmosphere with a ramp rate of 10 °C min^–1^ and a 2 min dwell time prior to each data acquisition at 10 °C intervals were used. Rietveld refinement was utilized using GSAS-II software to calculate the TaN crystal volume, from which CTE was derived using eq 5, where α is the CTE and *V* is volume.
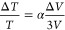
5

## Supporting Information Available

**Figure S1,** stress thickness product quantified using wafer flexure measurements following each process step with respect to the previous step and cumulatively; **Figure S2,** picosecond acoustic data of each electrode material and aluminum, used as the top reference layer, shown with indexing to denote the signature used to calculate the acoustic wave time delay; **Figure S3,** metastable phase *d*-spacing and peak normalized area with respect to sin^2^ ψ measured on devices fabricated with platinum, iridium, tantalum nitride, ruthenium, and tungsten top electrode materials, respectively; **Figure S4,** averaged and unwarped area detector X-ray diffraction patterns measured on samples with blanket electrodes; **Table S1,** high temperature powder X-ray diffraction derived Rietveld refinement data; **Table S2,** TaN linear coefficient of thermal expansion calculation data (PDF)
