# Supplementary material for: Electrode Elastic Modulus as the Dominant Factor in the Capping Effect in Ferroelectric Hafnium Zirconium Oxide Thin Films
Source: ACS Appl Mater Interfaces. 2024 Dec 3;16(50):69588–98. doi: 10.1021/acsami.4c15934 (PMC11660034; doi:10.1021/acsami.4c15934)
Supplement: Supplementary file 1 — am4c15934_si_001.pdf [file am4c15934_si_001.pdf]

Electrode Elastic Modulus as the Dominant Factor in the Capping Effect in Ferroelectric Hafnium Zirconium Oxide Thin Films

Megan K. Lenox,[1] Md Rafiqul Islam,[2] Md Shafkat Bin Hoque,[2] Chloe H. Skidmore,[3] Alejandro Salanova,[1,a)] Shelby S. Fields,[1,a)] Samantha T. Jaszewski,[1,b)] Jon-Paul Maria,[3] Patrick E. Hopkins,[1,2,4] and Jon F. Ihlefeld[1,5,]*

[1]Department of Materials Science and Engineering, University of Virginia, Charlottesville, Virginia 22904, United States

[2]Department of Mechanical and Aerospace Engineering, University of Virginia, Charlottesville, Virginia 22904, United States

[3]Department of Materials Science and Engineering, Pennsylvania State University, University Park, Pennsylvania 16802, United States

[4]Department of Physics, University of Virginia, Charlottesville, Virginia 22904, United States

[5]Charles L. Brown Department of Electrical and Computer Engineering, University of Virginia, Charlottesville, Virginia 22904, United States

[*] Electronic Mail: jihlefeld@virginia.edu

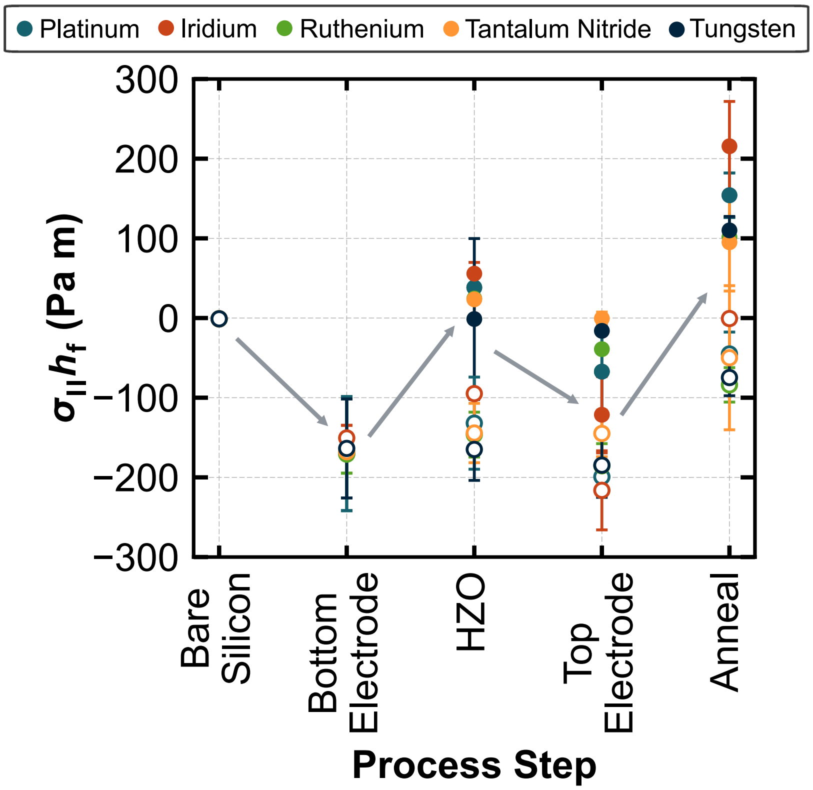

Figure S1: Stress thickness product quantified using wafer flexure measurements following each process step with respect to the previous step (closed circles) and cumulatively (open circles).

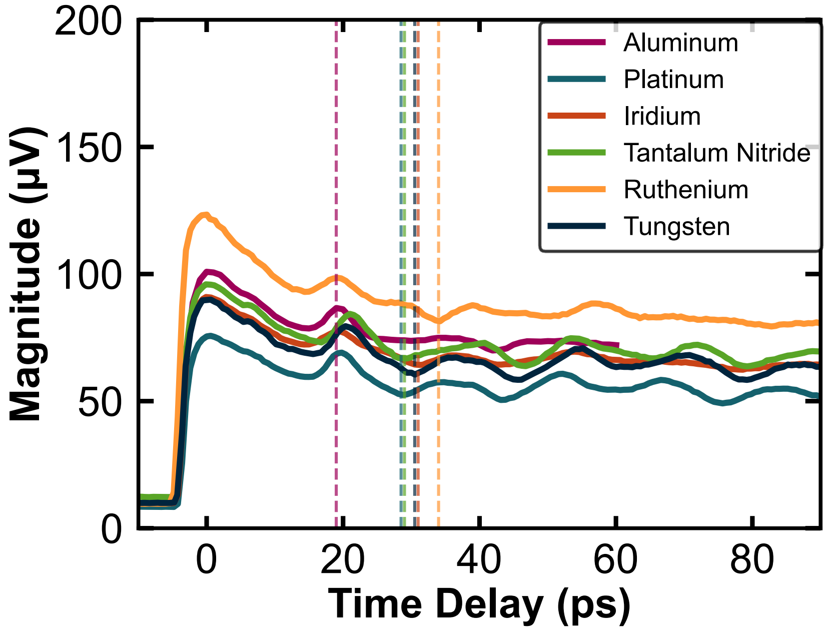

Figure S2: Picosecond acoustic data of each electrode material and aluminum, used as the top reference layer, shown with indexing to denote the signature used to calculate the acoustic wave time delay.

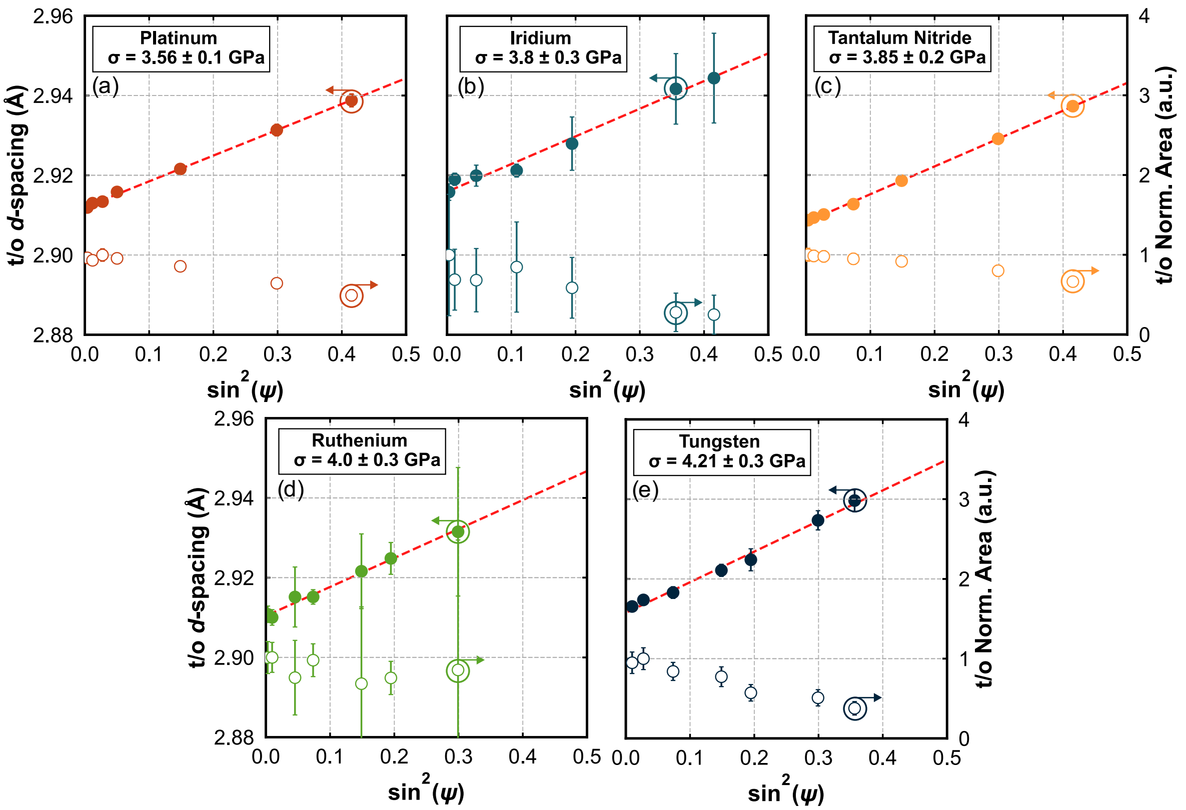

Figure S3: (a)-(e) Metastable phase *d*-spacing (closed point) and peak normalized area (open point) with respect to $\sin^2\psi$ measured on devices fabricated with platinum, iridium, tantalum nitride, ruthenium, and tungsten top electrode materials, respectively. The red dashed line indicates the linear fit used to calculate the biaxial stress in the HZO layer.

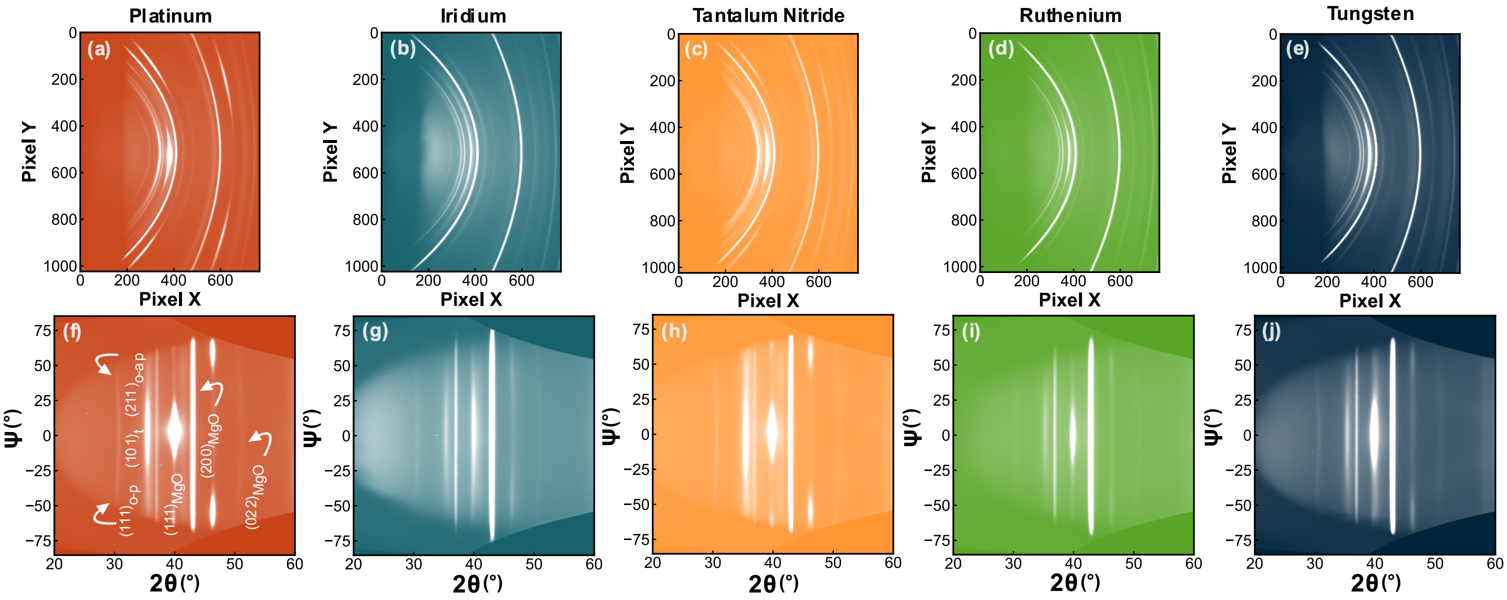

Figure S4: (a)-(e) Averaged and (f)-(j) unwarped area detector X-ray diffraction patterns measured on samples with blanket electrodes.

Table S1: High temperature powder X-ray diffraction derived Rietveld refinement data.

	No.	Rwp	Temperature (K)	Lattice Parameter, a (Å)	Lattice Parameter, a (Å) estimated standard deviation	Lattice Parameter, c (Å)	Lattice Parameter, c (Å) estimated standard deviation	Unit Cell Volume (Å^3)	Unit Cell Volume estimated standard deviation	Crystallite Size (um)	Crystallite Size estimated standard deviation
HTK1200N_Cu	0	14.68075509	928	5.205910721	0.000258409	2.91966746	8.12E-05	68.5263268	0.00567151	0.299270334	0.003894496
HTK1200N_Cu	1	14.96180059	918	5.20571822	0.000261274	2.919541788	8.21E-05	68.51830968	0.005733113	0.303664674	0.00405838
HTK1200N_Cu	2	14.87762768	908	5.205431665	0.000258206	2.919313806	8.13E-05	68.50541663	0.00566778	0.306379338	0.004162509
HTK1200N_Cu	3	14.93859638	898	5.205169798	0.000258623	2.919172203	8.13E-05	68.49520171	0.00567251	0.306064266	0.00418125
HTK1200N_Cu	4	15.0753645	888	5.204944503	0.000260362	2.918987043	8.21E-05	68.48492831	0.005715777	0.313292985	0.004208152
HTK1200N_Cu	5	15.11248582	878	5.204646785	0.000261126	2.918792218	8.22E-05	68.47252354	0.005727897	0.309991922	0.004212574
HTK1200N_Cu	6	15.28113806	868	5.204254337	0.000258999	2.918567409	8.15E-05	68.45692476	0.005678883	0.316373587	0.004384051
HTK1200N_Cu	7	15.43113019	858	5.204045961	0.000260105	2.918392411	8.21E-05	68.44733854	0.005708195	0.320012721	0.004494882
HTK1200N_Cu	8	15.415951	848	5.203858984	0.000259188	2.918223376	8.16E-05	68.43845589	0.005683838	0.322506808	0.004545608
HTK1200N_Cu	9	15.45735054	838	5.203549649	0.000259111	2.918070755	8.16E-05	68.42674087	0.005681265	0.325185764	0.004603736
HTK1200N_Cu	10	15.58851649	828	5.203147879	0.000259397	2.917771728	8.18E-05	68.4091638	0.005688581	0.329137911	0.004719632
HTK1200N_Cu	11	15.63081818	818	5.202982803	0.000259685	2.917642574	8.18E-05	68.40179525	0.005691902	0.328715405	0.004710254
HTK1200N_Cu	12	15.66142326	808	5.20263303	0.000259417	2.917417102	8.18E-05	68.38731357	0.005687395	0.330193438	0.00474756
HTK1200N_Cu	13	15.71769513	798	5.202347508	0.000260531	2.917164585	8.21E-05	68.37388892	0.005709962	0.329818587	0.004746244
HTK1200N_Cu	14	15.57663375	788	5.20207017	0.000256368	2.917014194	8.08E-05	68.36307452	0.005614964	0.333486851	0.004775489
HTK1200N_Cu	15	15.7502663	778	5.201840386	0.000258984	2.916804727	8.17E-05	68.3521266	0.005674942	0.333153689	0.00482127
HTK1200N_Cu	16	15.706328	768	5.201468201	0.00025952	2.916589805	8.17E-05	68.33731019	0.005683208	0.331370125	0.004772516
HTK1200N_Cu	17	15.74454209	758	5.201246788	0.000259846	2.916387521	8.18E-05	68.32675321	0.005690313	0.330752694	0.00477147
HTK1200N_Cu	18	15.7054123	748	5.200961307	0.000258628	2.916206821	8.14E-05	68.31501984	0.005661012	0.333928083	0.004803719
HTK1200N_Cu	19	15.68351709	738	5.200685595	0.000258975	2.915990935	8.15E-05	68.30272023	0.005667837	0.33195012	0.004763257
HTK1200N_Cu	20	15.91724339	728	5.200450197	0.00026184	2.915803421	8.25E-05	68.29214539	0.005731127	0.334468514	0.004881466
HTK1200N_Cu	21	16.02744137	718	5.200135393	0.000263361	2.91560901	8.31E-05	68.2793248	0.005766223	0.334160144	0.00490922
HTK1200N_Cu	22	15.98825979	708	5.1998509	0.000261562	2.91539328	8.25E-05	68.26680253	0.005725586	0.336977552	0.004974647
HTK1200N_Cu	23	15.88946954	698	5.199579165	0.000260637	2.915208559	8.21E-05	68.25534275	0.005702901	0.336135326	0.004935051
HTK1200N_Cu	24	15.80787316	688	5.19933212	0.000260832	2.914978633	8.21E-05	68.24347403	0.005705668	0.330703497	0.004762817
HTK1200N_Cu	25	15.75074977	678	5.198999486	0.000257009	2.914786039	8.10E-05	68.2302341	0.00562112	0.337252411	0.00490021
HTK1200N_Cu	26	15.60012366	668	5.198701832	0.000256136	2.914581493	8.07E-05	68.21763412	0.005603179	0.333263245	0.004743396
HTK1200N_Cu	27	15.55669506	658	5.198453446	0.000254529	2.914368138	8.02E-05	68.2061224	0.005565878	0.334677758	0.00475072
HTK1200N_Cu	28	15.56868967	648	5.19816418	0.000256249	2.914200679	8.06E-05	68.19461333	0.005604317	0.334429447	0.004752019
HTK1200N_Cu	29	15.83510209	638	5.197974931	0.000261155	2.914029887	8.22E-05	68.18565153	0.005709397	0.331369181	0.004775455
HTK1200N_Cu	30	15.72038631	628	5.197664045	0.000258186	2.913802407	8.13E-05	68.17217333	0.005643997	0.332501832	0.004759264
HTK1200N_Cu	31	15.76291986	618	5.19733924	0.000257369	2.913586271	8.11E-05	68.15859723	0.005624443	0.335885673	0.004865757
HTK1200N_Cu	32	15.79852869	608	5.197174751	0.000259139	2.913438801	8.15E-05	68.15083342	0.005660299	0.331896205	0.004771034
HTK1200N_Cu	33	15.69981698	598	5.196913049	0.000255983	2.913276165	8.05E-05	68.14016622	0.005591485	0.337192799	0.004867479
HTK1200N_Cu	34	15.63286868	588	5.196535009	0.000256638	2.913014729	8.08E-05	68.12413912	0.005607529	0.331878854	0.004705215
HTK1200N_Cu	35	15.57131272	578	5.196307619	0.000256329	2.912865825	8.06E-05	68.11469532	0.005598694	0.331175598	0.004681246
HTK1200N_Cu	36	15.54999637	568	5.196022753	0.000256918	2.912660374	8.07E-05	68.10242356	0.005609629	0.327819233	0.004602012
HTK1200N_Cu	37	15.58818607	558	5.195816112	0.000256738	2.912500269	8.07E-05	68.0932637	0.005603639	0.329781226	0.004658021
HTK1200N_Cu	38	15.46734452	548	5.195602371	0.000253583	2.912324574	7.98E-05	68.08355417	0.005536372	0.331893045	0.004657389
HTK1200N_Cu	39	15.59865293	538	5.195205588	0.000256299	2.912080009	8.06E-05	68.06743912	0.005592108	0.330730704	0.004683043
HTK1200N_Cu	40	15.66361551	528	5.195084814	0.00025756	2.91194743	8.11E-05	68.06117561	0.005624033	0.331449959	0.004710355
HTK1200N_Cu	41	15.46881137	518	5.194877202	0.00025508	2.911766112	8.02E-05	68.0514982	0.005568809	0.331176377	0.0046479
HTK1200N_Cu	42	15.64638063	508	5.194597289	0.000258316	2.9115839	8.12E-05	68.03990681	0.005634656	0.327211756	0.0046318
HTK1200N_Cu	43	15.46933302	498	5.194296953	0.000255656	2.911402682	8.04E-05	68.02780495	0.005578404	0.327600018	0.004575621
HTK1200N_Cu	44	15.34396842	488	5.194079271	0.000253166	2.91122239	7.95E-05	68.01789093	0.005521526	0.328426691	0.004563336
HTK1200N_Cu	45	15.66748674	478	5.193868567	0.000259	2.911066272	8.14E-05	68.00872532	0.005649897	0.32802498	0.004650981
HTK1200N_Cu	46	15.61865934	468	5.193591312	0.000258875	2.910877633	8.14E-05	67.99705821	0.005645075	0.324951129	0.004578032
HTK1200N_Cu	47	15.42848725	458	5.193302727	0.000254766	2.910688966	8.02E-05	67.98509513	0.005557177	0.327802183	0.004571505
HTK1200N_Cu	48	15.52934974	448	5.19319465	0.00025664	2.910559625	8.06E-05	67.97924461	0.005595037	0.327972305	0.004606792
HTK1200N_Cu	49	15.61309325	438	5.19288256	0.00025905	2.910363399	8.15E-05	67.96649178	0.005649367	0.325723582	0.00459138
HTK1200N_Cu	50	15.47329666	428	5.192680962	0.000256689	2.910215906	8.06E-05	67.95777051	0.005595165	0.325494055	0.004548829
HTK1200N_Cu	51	15.43431715	418	5.192414738	0.000256098	2.910015322	8.06E-05	67.94611899	0.005581934	0.323516313	0.004497193
HTK1200N_Cu	52	15.38816736	408	5.192227902	0.000255825	2.909882628	8.03E-05	67.93813129	0.005571492	0.322911886	0.004477715
HTK1200N_Cu	53	15.33360064	398	5.191967096	0.000254614	2.909716298	8.00E-05	67.92742339	0.005547087	0.324947	0.004499544
HTK1200N_Cu	54	15.3922704	388	5.191750407	0.000255095	2.909549527	8.01E-05	67.91786061	0.005554712	0.325402116	0.004521628
HTK1200N_Cu	55	15.10071122	378	5.191578973	0.000250984	2.909405508	7.88E-05	67.91001368	0.005466545	0.323034694	0.004399264
HTK1200N_Cu	56	15.22415208	368	5.191291789	0.000254342	2.90922868	7.99E-05	67.89837371	0.005541007	0.320740546	0.004399152
HTK1200N_Cu	57	15.31401999	358	5.19107837	0.000255309	2.909071583	8.01E-05	67.88912491	0.005558068	0.323207458	0.004471964
HTK1200N_Cu	58	15.14431199	348	5.190924126	0.00025301	2.908925939	7.95E-05	67.88169186	0.005510773	0.320535787	0.004372864
HTK1200N_Cu	59	15.20918424	338	5.190572804	0.000253438	2.908722469	7.95E-05	67.86775621	0.005513814	0.322546054	0.00444042
HTK1200N_Cu	60	15.3603882	328	5.190533029	0.000257418	2.908624303	8.07E-05	67.86442566	0.005598698	0.318662705	0.00440047
HTK1200N_Cu	61	15.0876337	318	5.190234638	0.000254151	2.908447402	7.97E-05	67.8524962	0.005530797	0.319380837	0.004354032
HTK1200N_Cu	62	15.12116248	313	5.190303864	0.00025322	2.90842735	7.94E-05	67.85383837	0.005509896	0.32054481	0.004386214

Table S2: TaN linear coefficient of thermal expansion calculation data

Temperature (K)	Volume (Å^3)	$\Delta V/3V_o$	CTE $\Delta L/\Delta LT$ (K^{-1}) in 50 K intervals	CTE ($x10^{-6}$ K^{-1})
313	67.8537473	0.00E+00		
318	67.8524392	-6.43E-06	3.84E-06	3.8364
328	67.86282993	4.46E-05	4.56E-06	4.5588
338	67.86771483	6.86E-05	4.34E-06	4.3437
348	67.88158831	1.37E-04	4.77E-06	4.7703
358	67.88762999	1.66E-04	4.51E-06	4.5092
368	67.89669895	2.11E-04	4.64E-06	4.6433
378	67.90811911	2.67E-04	4.84E-06	4.8428
388	67.91588246	3.05E-04	4.63E-06	4.6313
398	67.92597713	3.55E-04	5.03E-06	5.0320
408	67.93666195	4.07E-04	4.70E-06	4.7009
418	67.94434172	4.45E-04	4.81E-06	4.8125
428	67.95584699	5.02E-04	5.11E-06	5.1080
438	67.96468375	5.45E-04	4.95E-06	4.9502
448	67.97734112	6.07E-04	5.03E-06	5.0304
458	67.98339247	6.37E-04	4.98E-06	4.9779
468	67.99544043	6.96E-04	5.44E-06	5.4395
478	68.00672849	7.52E-04	5.27E-06	5.2671
488	68.01630748	7.99E-04	4.84E-06	4.8398
498	68.02612372	8.47E-04	5.35E-06	5.3521
508	68.0381399	9.06E-04	5.33E-06	5.3294
518	68.04982801	9.63E-04	5.13E-06	5.1308
528	68.05977057	1.01E-03	5.13E-06	5.1343
538	68.06583974	1.04E-03	5.07E-06	5.0659
548	68.081676	1.12E-03	5.90E-06	5.9045
558	68.09121492	1.17E-03	5.42E-06	5.4243
568	68.10080523	1.21E-03	5.34E-06	5.3447
578	68.11253707	1.27E-03	5.63E-06	5.6323
588	68.12164331	1.32E-03	5.80E-06	5.7966
598	68.13795518	1.40E-03	5.79E-06	5.7921
608	68.14792674	1.45E-03	5.34E-06	5.3408
618	68.15649319	1.49E-03	5.51E-06	5.5115
628	68.16959681	1.55E-03	5.83E-06	5.8277
638	68.18333452	1.62E-03	5.87E-06	5.8733
648	68.19238556	1.66E-03	5.67E-06	5.6731
658	68.20318587	1.72E-03	5.88E-06	5.8772
668	68.21524272	1.78E-03	6.03E-06	6.0283
678	68.22767098	1.84E-03	6.10E-06	6.1022
688	68.24133139	1.90E-03	5.93E-06	5.9331
698	68.25262394	1.96E-03	5.85E-06	5.8457
708	68.26416741	2.02E-03	5.82E-06	5.8232
718	68.27681394	2.08E-03	5.75E-06	5.7452
728	68.28977347	2.14E-03	5.95E-06	5.9527
738	68.30013558	2.19E-03	5.83E-06	5.8333
748	68.31272849	2.25E-03	5.82E-06	5.8226
758	68.32374635	2.31E-03	5.91E-06	5.9122
768	68.33433759	2.36E-03	6.17E-06	6.1740
778	68.34951828	2.44E-03	5.92E-06	5.9182
788	68.36101937	2.49E-03	6.14E-06	6.1424
798	68.37125133	2.54E-03	6.17E-06	6.1654
808	68.38493774	2.61E-03	6.05E-06	6.0538
818	68.39915305	2.68E-03	5.70E-06	5.6950
828	68.40662107	2.72E-03	5.85E-06	5.8503
838	68.42453908	2.80E-03	6.31E-06	6.3113
848	68.43632168	2.86E-03	5.76E-06	5.7558
858	68.44519024	2.91E-03	5.63E-06	5.6295
868	68.4544951	2.95E-03	5.98E-06	5.9765
878	68.47060606	3.03E-03	5.87E-06	5.8717
888	68.48370545	3.09E-03	null	null
898	68.49483886	3.15E-03	null	null
908	68.50507891	3.20E-03	null	null
918	68.51818546	3.26E-03	null	null
928	68.52621054	3.30E-03	null	null

AUTHOR INFORMATION

Corresponding Author

*Jon Ihlefeld, Electronic Mail: jihlefeld@virginia.edu

Present Addresses

a) Present Address: U.S. Naval Research Laboratory, Washington, D.C. 20375, United States

b) Present Address: Sandia National Laboratories, Albuquerque, New Mexico 87185, United States
